# Supplementary material for: Antioxidant Activity and Sensory Quality of Bacon
Source: Foods. 2022 Jan 17;11(2):236. doi: 10.3390/foods11020236 (PMC8774322; doi:10.3390/foods11020236)

**Supplementary Figure S1. Sensory panel evaluation of bacon produced using liquid smoke prepared from different woods.** CS, commercial liquid smoke; CE, *Cupressus funebris* Endl; AL, *Armeniaca vulgaris* Lam; DT, *Diospyros kaki* Thunb; PL, *Punica granatum* L.; ZM, *Ziziphus jujuba Mill.*

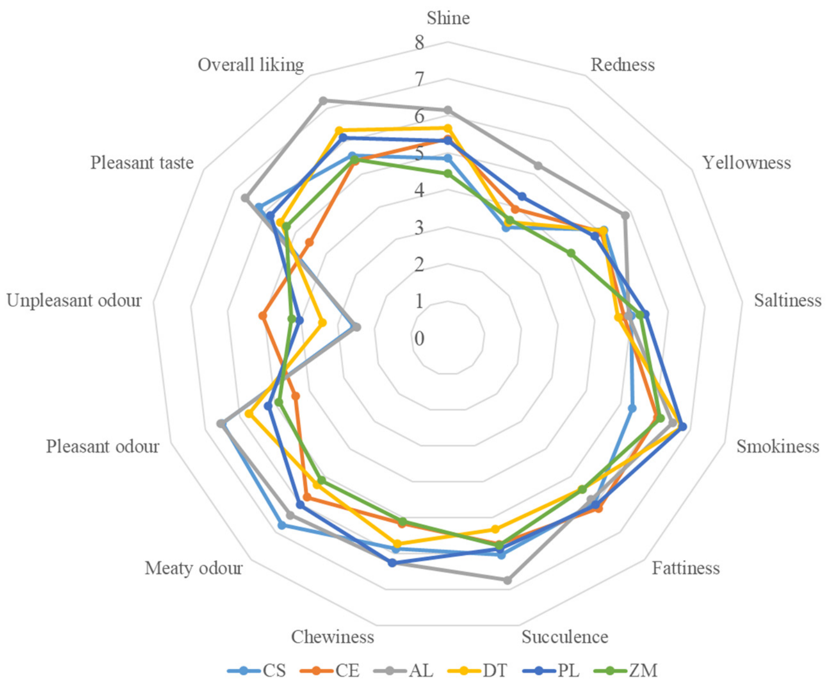

Supplement: Supplementary file 1 [file foods-11-00236-s001.zip › foods-1462103-supplementary.pdf]
